# Bhopal, Dunsmuir and TRPA1: what they have taught us about nociception

**DOI:** 10.3389/falgy.2025.1609137

**Published:** 2025-09-29

**Authors:** Dennis J. Shusterman, Andrew G. Salmon

**Affiliations:** 1 Upper Airway Biology Laboratory, Division of Occupational, Environmental and Climate Medicine, University of California, San Francisco, CA, United States; 2 Office of Environmental Health Hazard Assessment, California Environmental Protection Agency, Oakland, CA, United States

**Keywords:** TRPA1 activation, irritant-induced asthma, Bhopal accidental release, railroad accident, pesticides: adverse effects

## Abstract

On separate occasions nearly a decade apart, two large-scale accidental releases of industrial chemicals exposed substantial “bystander” (non-worker) populations to highly toxic air pollutants. The first of these events, occurring in Bhopal, India in 1984, generated worldwide attention and concern given its geographic scope and significant lethality. The second incident, occurring in Dunsmuir, CA in 1991 – while less publicized – yielded new insights into the pathogenesis of irritant-induced asthma. Linking these events is the fact that the toxicants involved – methyl isocyanate (MIC) in Bhopal and methyl isothiocyanate (MITC) in Dunsmuir – preferentially bind to the same TRPA1 nociceptive ion channel. This review examines each of these exposure events, including their mechanistic implications for anticipating (and potentially preventing) future long-term health effects from accidental chemical exposures.

## Introduction

Occupational and environmental toxicology has – in part – advanced by extrapolating health effects seen in heavily exposed occupational cohorts (individuals in the workplace) to less heavily exposed environmental cohorts (i.e., non-workers in adjacent areas). Emphasizing this fact, the effects of workplace exposures on workers are sometimes referred to as “inside the fence” and bystander exposures “outside the fence.” Notwithstanding this graphic model, exceptions do occur, with so-called “fugitive air pollutants” being a case in point.

Most fugitive emissions involve relatively innocuous air pollutants originating in pulp mills, concentrated animal feeding operations, water and waste treatment plants, landfills, and geothermal energy sources. These fugitive emissions may involve highly odorous compounds with substantial “warning properties” (i.e., highly potent *odorants*). In this review, by contrast, we consider two examples from the opposite end of the exposure spectrum (i.e., highly potent *irritants*). These are compounds capable of irritating one's mucous membranes and respiratory tract at concentrations that are barely perceptible based upon their odor alone.

Of the fugitive releases alluded to above, the first – in Bhopal, India in 1984 – produced health effects spanning from acute eye, nose, throat and chest irritation at one extreme, to frank pulmonary edema and mortality at the other. In the second release – a railroad train derailment in Dunsmuir, CA in 1991 – acute mucous membrane irritation (eye, nose, throat and chest) resolved relatively promptly in most cases, but were followed by persistent asthma-like symptoms in a subset of those exposed. While mass exposure incidents are by no means rare, these two incidents were linked by a subsequent molecular biology breakthrough: the sequencing of a shared irritant receptor (“ion channel”) in 2004. This coincidence provides a framework for potentially integrating our understanding of the health impacts of a wide range of exposure scenarios in the future.

**Figure 1:**
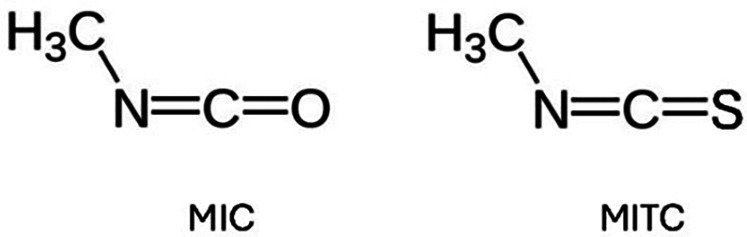
Molecular structures of methyl isocyanate (MIC) and methyl isothiocyanate (MITC).

Structurally, the toxicants ultimately implicated in these two incidents were methyl isocyanate or “MIC” (CH_3_-N = C = O) in Bhopal, and methyl iso*thio*cyanate or “MITC” (CH_3_-N = C = S) in Dunsmuir, molecules that differ by only a single atom (Figure 1). Put succinctly, MITC is the *sulfur analog* of MIC. Not surprisingly, the two compounds have overlapping biochemical and physiologic impacts. Although the circumstances and precise chemistries of the two releases differed, the two exposure incidents were otherwise remarkable similar, shedding light on the importance of molecular biology in understanding toxicologic phenomena.

The research breakthrough alluded to above involved the cloning of the shared molecular receptor for MIC and MITC – initially referred to as “ANK-TM1” and subsequently renamed “TRPA1” or “transient receptor potential ankryn1” (2). TRPA1 provided a significant addition to a series of tetrameric trans-membrane (TRP) ion channels involved in sensing temperature, pH, and (importantly) noxious chemicals. Earlier-identified members of this receptor family included the capsaicin receptor “TRPV1” [V for vanilloid] and the menthol receptor “TRPM8” [M for melastatin], receptors that are physiologically important in their own right (3–5). Despite many similarities amongst these receptors, TRPA1 distinguishes itself from the others by exhibiting an extremely wide range of chemical ligands with which it binds (and is activated). The collection of substances that bind to (and react with) TRPA1 – in fact – encompasses multiple identified air pollutants, reactive industrial chemicals, and household cleaning products, rendering TRPA1 (and potentially its antagonists) of outsized importance in occupational and environmental medicine, allergy, and public health in general (6, 7).

**Figure 2:**
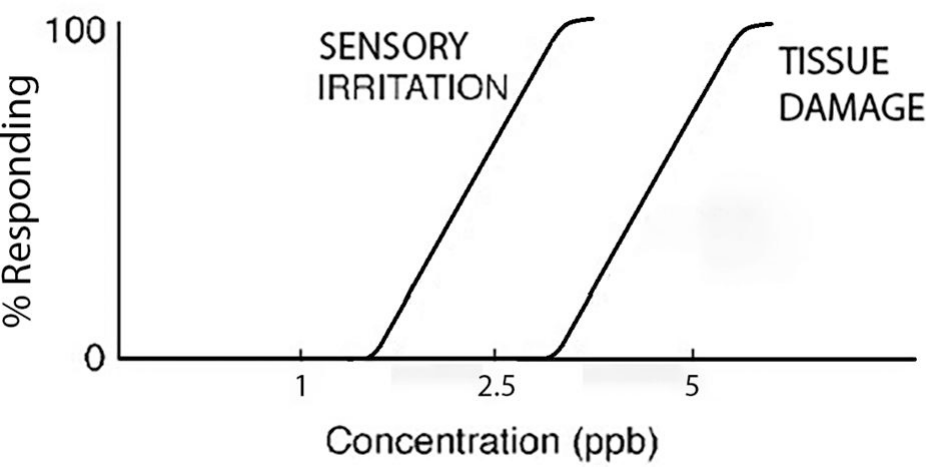
Hypothetical dose-response relationship comparing sensory irritation and tissue damage for a given toxicant. [Image adapted from (1)].

Implicit in this discussion is a major underlying reality of toxicologic dose-response relationships. Specifically, receptor-mediated biological effects tend to manifest at lower exposure levels than do more robust toxicologic effects. Put in other terms, frank tissue damage from high-dose exposures may mask the more subtle messages involved in receptor threshold stimulation for a given target organ (Figure 2). For this reason, the two exposure scenarios considered here are examined side-by-side to explore the dose-response window between superficial (sensory) irritation and potentially devastating tissue damage. To assist us in this process, the reported physical and sensory attributes of MIC and MITC are tabulated in Table 1.

**Table 1:** Physical and psychophysical properties of MIC and MITC.

Compound	MW (g/mol)	Boiling point (^o^ C)	Odor threshold (ppm/1 sniff)	Irritant threshold (ppm/condition)	Water solubility (g/L)
MIC	57.05	39	2.1	1.3 (sensory) 1.9 (pulmonary)	6–10
MITC	73.12	117	1.7	3.3 (4 min – eye) 1.9 (10 min – eye)	8.2

Sources: MIC-Ferguson et al. (8); MITC-Cain et al. (9).

## The exposure incidents – similarities and differences

For this review, we consulted publications that were familiar to the authors by virtue of our prior public health duties (as well as related nociceptive biology) related to the [Bhopal and Dunsmuir] incidents. We then elaborated on those sources by conducting structured PubMed searches addressing key sub-questions. For similarities and differences between the two exposure incidents, see below (and Appendix A).

**Figure 3:**
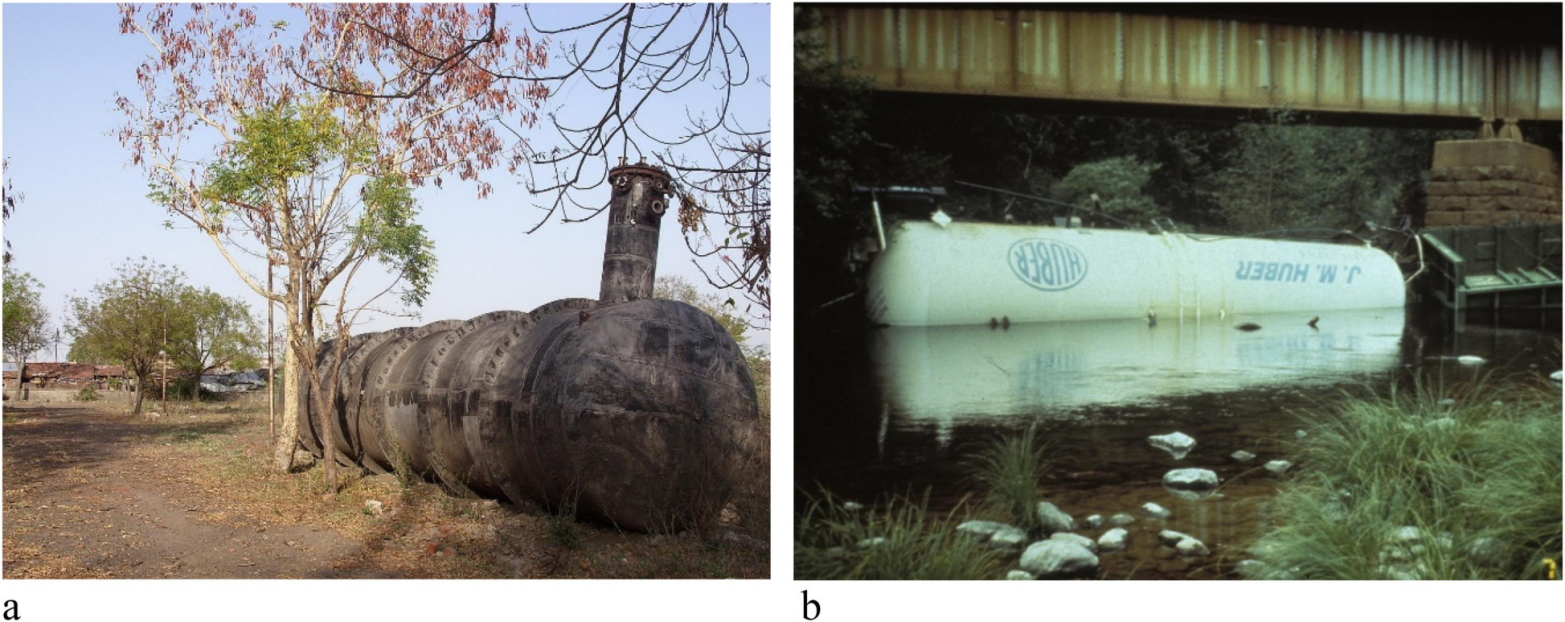
*Frame a*: MIC-containing tank that ruptured in bhopal. *Frame b***:** derailed Metam Sodium tank car at Dunsmuir [Credits: **(a)** Julian Nyča – Creative Commons; **(b)** Jay Mather Photography].

At Bhopal, *preformed* MIC was stored in a large tank onsite, where it was used in the formulation of carbamate pesticides. This stored MIC was ejected into the atmosphere after water was mistakenly introduced into the tank in which it was stored, thereby triggering an exothermic reaction and breaching the tank's seals (Figure 3a). By contrast, the MITC released in Dunsmuir was generated *in situ* by the hydrolysis of the soil fumigant Metam Sodium (sodium methylcarbamodithioate). Consistent with the fact that the Dunsmuir spill involved a railroad car derailment on a river overpass, hydrolysis occurred as the concentrated pesticide solution leaked from the fallen tank car, reacting with the water of the Sacramento River (Figure 3b). In both cases, crucial safety precautions had been ignored prior to the releases (10, 11). Details of these two releases (including supporting data from smaller-scale incidents) appear below, followed by a discussion of MIC's and MITC's interaction with the TRPA1 receptor. Finally, consideration is given to the wide range of additional TRPA1 ligands that have since been documented, and their far-reaching consequences for public health and environmental toxicology.

## Bhopal

The run-away exothermic reaction at the Union Carbide plant in Bhopal that occurred on December 2–3, 1984 is estimated to have released about 40 tons of methyl isocyanate over a period of 90 min (12). This assumed the form of a heavier-than-air vapor (referred to by observers as a “ground-hugging fog”) which spread across the surrounding area, including various settlement areas (many with sub-standard housing). This resulted in potential exposures to more than 200,000 people, with the intensity of exposure correlating [roughly] inversely with distance from the plant (13, 14). Subsequent analyses included meteorological data (e.g., wind direction and speed), and predicted a more detailed version of the exposures in various affected areas (12). This latter model predicted exposures exceeding 25 ppm in the most severely affected zones, 8–19 ppm (termed “badly affected”), 1.5–4 ppm (“moderately affected”) and less than 1 ppm (“mildly affected”) (12).

**Figure 4:**
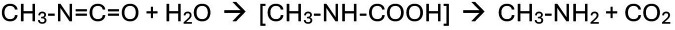
Hydrolysis products of methyl isocyanate (MIC), initially including methyl carbamic acid as a transient intermediate in the formation of methylamine and CO_2._subsequent reaction products may include dimethylurea and trimethyl biuret.

Dynamically speaking, in the vapor phase, MIC reacts relatively slowly with water vapor. Thus, regardless of the relative humidity of the air at the time, the gaseous toxicant to which people at Bhopal were exposed on an airborne basis would have been primarily MIC. Reaction with liquid water on the other hand (e.g., on the surface of exposed tissues such as the eye and respiratory tract epithelia), leads to rapid hydrolysis, initially producing methylamine and CO_2_. Subsequent reaction products may include dimethylurea and trimethyl biuret (15) (Figure 4). Of note, MIC has a very faint odor which may (or may not) be perceptible at its minimum irritant concentration (in the region of∼1 ppm; Table 1). Not surprisingly, considerable concern was expressed in scientific circles post-incident given the combination of severe outcomes at Bhopal and the fact that the published scientific literature on MIC toxicology was scarce at the time.

Various contemporary reports described around 2,500 deaths in the immediate aftermath of the incident (up to 72 h), with fatalities continuing at a substantial but declining rate thereafter. The government of Madhya Pradesh has reportedly stated that mortality associated with exposure to MIC had reached 3,598 by November 1989, and more than 6,000 by 1994 (16). Initial reports of symptoms suffered by those exposed to MIC identified the eyes and respiratory system as the predominant targets.

With regard to the eyes, on the first day after the disaster, an estimated 8,000 people were seen as outpatients or inpatients by medical providers at Gandhi Medical College. Those reporting eye injury reported severe pain and irritation and were found to suffer from superficial interpalpebral erosion of the cornea (17). The risk of acute corneal damage was generally correlated with severity of exposure. Other ocular pathology included dilated retinal vessels and hemorrhages seen among some more heavily exposed subjects in areas where deaths were noted.

The corneal erosions observed in the immediate event's aftermath, in general, healed without noticeable scarring within three months (15). Follow-up studies of health effects in cohorts exposed to MIC at Bhopal found increased eye watering (18) and – three years later – a threefold excess of eyelid inflammation, twofold increase of new cataracts, and finally loss of visual acuity among the more severely exposed clusters (19). These authors also reported a case-referent analysis confirming an excess of recent eye infections and irritant symptoms in people exposed to MIC. Andersson et al. (19) considered that “In its response to MIC the eye could be considered a sentinel organ for more general phenomena in the rest of the body”.

By contrast to transient ocular irritation, the presence of severe respiratory symptoms could be considered potentially more ominous (13). These symptoms included chest irritation, substernal chest pain, cough, choking and shortness of breath (13, 16). At Bhopal, respiratory irritation was often followed by nausea, convulsions, coma, and death. Andersson et al. (13) noted that “All these effect groups occurred at characteristic and different exposure levels: in particular, sensory and neurological responses were noticed at relatively low doses, the eye damage was apparent at intermediate exposures, and the more obvious and life-threatening manifestations of pulmonary damage appeared at higher dose levels.”

The highest mortality (due to respiratory failure) was seen among the sub-population living closest to (and downwind of) the plant, particularly those in substandard housing lacking sealable windows and doors. Symptoms and autopsy findings in the few days after the release of MIC were consistent with pulmonary edema/chemical pneumonitis (20). Mortality rates subsequently declined with the passage of time (21).

Many survivors who complained of respiratory symptoms showed long-term respiratory impacts including declines in lung function (16). Pulmonary function decline in individuals previously hospitalized after exposure to MIC was observed subsequently (1–7 years after exposure) and were associated with accumulation of inflammatory cells (macrophages, neutrophils and lymphocytes) in the lung. The extent of these changes was correlated with the estimated severity of exposure (22). Similar deficits in lung function (particularly small airway disease), and reported symptoms (dyspnea, phlegm, cough, wheeze) were reported ten years after the disaster with frequency and severity greatest among those closer to the site of the accident (23). Readers interested in the longitudinal analysis of Bhopal's MIC-exposed cohort are encouraged to access Appendix B, which indexes both the health surveys and [physical and laboratory] examinations conducted periodically through 2016.

Only one study had been published of MIC toxicology *in vivo* in animals at the time of the Bhopal release. This rodent study documented dose-related disruptions in breathing patterns in rats, mice, rabbits and guinea pigs at lower exposure levels, and pulmonary edema and death at higher levels (24). Fueled by this relative publication void (and the scale and gravity of health effects at Bhopal), additional research was soon completed regarding MIC's effects. Rats were exposed to fluctuating MIC levels between 0.1 and 1.0 mg/L over durations between 15 min and 1 h (25). Steady state 2-hour exposures of rats to between 5 and 65 ppm MIC were also reported (26, 27). In these studies, at low levels, alterations in respiratory behavior indicative of both sensory and pulmonary irritation were noted. Ocular lesions (punctate corneal erosions) were reported at intermediate exposures. Higher exposures produced pulmonary edema and death in the majority of animals. Subsequent studies by these authors further explored histopathology, dose response and longer-term effects including delayed mortality and recovery (28, 29). These studies showed broadly similar effects in rats to those seen in exposed humans at the time of the accident and subsequently.

In 1987 a series of large-scale studies in both rats and mice, conducted by the (US) National Toxicology Program (NTP), was published under the lead author Bucher. Animals were exposed by inhalation for 2 h to MIC at 0, 10, 20 or 30 ppm in sufficient numbers to allow periodic examination of survivors at 7 intervals (between 0 and 91 days post-exposure). At 20 or 30 ppm, animals began dying between 15 and 18 h post-exposure, with a second wave occurring 8–10 days later, and with most exhibiting “severe respiratory distress” prior to expiring (30). Dose-related lung weight increases were observed, but no systematic changes in the brain, liver, kidney, thymus, testis or spleen. Severe necrosis and epithelial damage were reported in the trachea and main bronchi of exposed rats (31) and mice (32). Necrosis and erosion were also observed in the upper respiratory tract of rats and mice (32, 33), but unlike the lower respiratory tract, these tissues regenerated to normal by 90 days following exposure.

These academically based MIC studies apparently motivated industry to share their backlog of information. At Union Carbide, reports describing toxicology studies of rats, mice and guinea pigs exposed to MIC were made available soon after the Bhopal disaster (34–38). These studies confirmed and extended the findings of severe effects on the lungs of rodents reported by the NTP investigators. They also showed greater sensitivity in guinea pigs. Cause of death during or immediately following exposure appeared to be severe obstruction of airways by mucus and cellular debris resulting in hypoxia and asphyxia. Examination of animals at longer times after exposure (39) showed persistent pulmonary congestion, fibrosis and inflammatory changes which, in rats which survived longer, showed signs of repair and regeneration of pulmonary and epithelial tissue.

The above reports of inhalation toxicity studies with MIC describe a slowed breathing pattern in exposed animals. This is a typical response in rodents, as documented by Alarie (40), where he defines “respiratory irritation” as a combination of sensory and pulmonary irritation, with subsequent reflex respiratory slowing. Two specific investigations of this phenomenon were reported, in MIC exposed mice (8, 41). An RD_50_ (concentration of MIC capable of slowing respiration by 50%) of 1.3 ppm was measured, indicating that MIC is a potent sensory irritant. Use of a tracheal cannula to expose mice to MIC (to observe direct irritation of the lower respiratory tract only) produced an RD_50_ TC value only 1.5 times higher, indicating that MIC is best described as a respiratory irritant, affecting both exposed mucous membranes and the lungs. A similar experiment by James et al. (41) observed an RD_50_ of 1.9 ppm, consistent with the Ferguson's observations.

Some investigators have also examined the possible toxicological impacts of the hydrolysis products of MIC, methylamine and dimethylurea (42–44). Although methylamine was not entirely without effect following inhalation, the authors concluded that the observed impacts of MIC exposure were due to the parent isocyanate rather than its hydrolysis products.

## Dunsmuir

The second major exposure incident we are considering has been referred to as “California's largest hazardous chemical spill” (*Wikipedia*, 10/2/2024). It occurred late on the night of July 14, 1991 with the derailment of a freight train crossing a remote overpass on the upper reaches of the Sacramento River. The spill itself resulted in 19,500 gallons of the soil fumigant, Metam Sodium (sodium methylcarbamodithioate), being released 40 miles upstream of Lake Shasta and 6 miles north of the town of Dunsmuir (population 2,129 per 1990 US Census).

**Figure 5:**
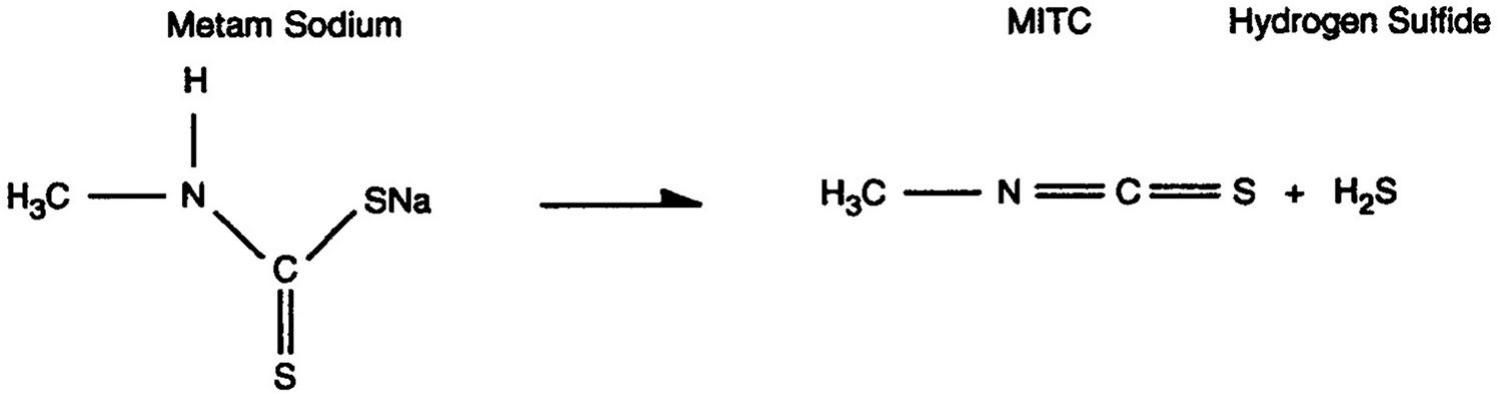
Formation of methyl isothiocyanate (MITC) by the reaction of metam sodium with water.

The pesticide in question was being transported in a single-walled tank car and had not been classified by the US Department of Transportation as a hazardous substance (the rationale being that it had *limited direct biological reactivity in its concentrated form*). Metam Sodium breaks down by hydrolysis when it is diluted in water (augmented during the day by photolysis), yielding as principal breakdown products methyl isothiocyanate (MITC) and hydrogen sulfide (H_2_S). Ironically, this scenario resulted in subacute MITC exposure to residents near the river over the next several days, as the pesticide in river water slowly traversed southward toward Shasta Lake through a recreational area known for its scenic beauty and prime trout fishing (Figure 5).

By the morning following the spill, the chemical contents of the leaking tank car were identified, the magnitude of the pesticide leak recognized, and acknowledgment made that the location of the derailment (the “Cantara Loop”) had in fact been the site of at least one previous railroad car derailment. Local residents, railroad and emergency response workers began to report a variety of symptoms, and by the afternoon of July 15 an emergency shelter was set up in an unaffected area of Dunsmuir. In addition to reporting irritative symptoms, several evacuees mentioned faint odors of “horseradish” or “rotten eggs,” and at least one reported having felt “tingling of the skin” when he approached the river (reminiscent of reports of abnormal skin sensations among laboratory workers exposed to MITC vapor).

It would be two more days before the leading edge of the spill reached Lake Shasta via the river, with MITC vapor (plus other minor breakdown products) being emitted on a continuous basis from the contaminated water, and diurnally shifting wind directions within the steep-walled river canyon prolonging the duration of potential human exposures. Although direct measurements of airborne MITC vapor did not start until 2 days after the spill, based on retrospective data, modeled peak airborne concentrations are thought to have reached as high as 1.6 ppm (45).

Emergency response representatives from more than 35 state, local, and federal agencies were involved in the response and were conspicuous along the Sacramento River corridor for some weeks to come (46). Besides eliciting symptom complaints among local residents and workers, the spill produced significant and widespread ecological impacts, including the deaths of hundreds of thousands of native trout (and other vertebrates), loss of trees and shrubs bordering the river, and near-complete elimination of numerous species of aqueous plants and invertebrates that called the river home and constituted essential members of the local food chain (47, 48).

With regard to human health, among the numerous responding agencies, representatives of the California Department of Health Services (CDHS) and the California Environmental Protection Agency (Cal/EPA) worked with local hospitals and county health departments (Siskiyou and Shasta) to provide health risk information to medical providers and local residents, help various agencies prioritize resources, and analyze symptom complaint data. A standardized symptom questionnaire was drafted by State personnel, distributed via local public health departments and local physicians, and used to abstract data both from local individuals and from the two nearby hospital emergency departments. A total of seven patients (three with pre-existing obstructive respiratory conditions) were admitted to hospitals as in-patients due to spill-related health effects (45). Over the 5-week period following the spill, a cumulative total of 705 complainants (71% of whom lived in Dunsmuir) reported acute symptoms including headache, eye, nose and throat irritation, nausea, and chest tightness/shortness-of-breath [see Table 2; (50)]. In addition to the above acute response, CDHS responded to delayed complaints of skin rashes among local inmates at a firefighting camp who were pressed into service removing dead fish from the river (51, 52).

**Table 2:** Prevalence of selected self-reported symptoms subsequent to exposure.

Symptoms	Bhopal (MIC) (49)	Dunsmuir (MITC) (50)
Number of respondents	978	705
Timing of survey	<2 days	<5 wks.
Mucous membranes	(% Reporting symptom)	
Sensory irritation (eye)	86	48
Sensory irritation (throat)	46	42
Sensory irritation (nasal)	*	23
Gastrointestinal	(% Reporting symptom)	
Nausea	52	48
Diarrhea	13	25
Abdominal pain	19	19
Respiratory	(% Reporting symptom)	
Shortness of breath	99	27
Chest tightness	*	22
Cough	95	14
Wheeze	*	12

Data source(s): Bhopal: Misra et al. (49); Dunsmuir: Kreutzer et al. (50).

* - Not recorded.

**Figure 6:**
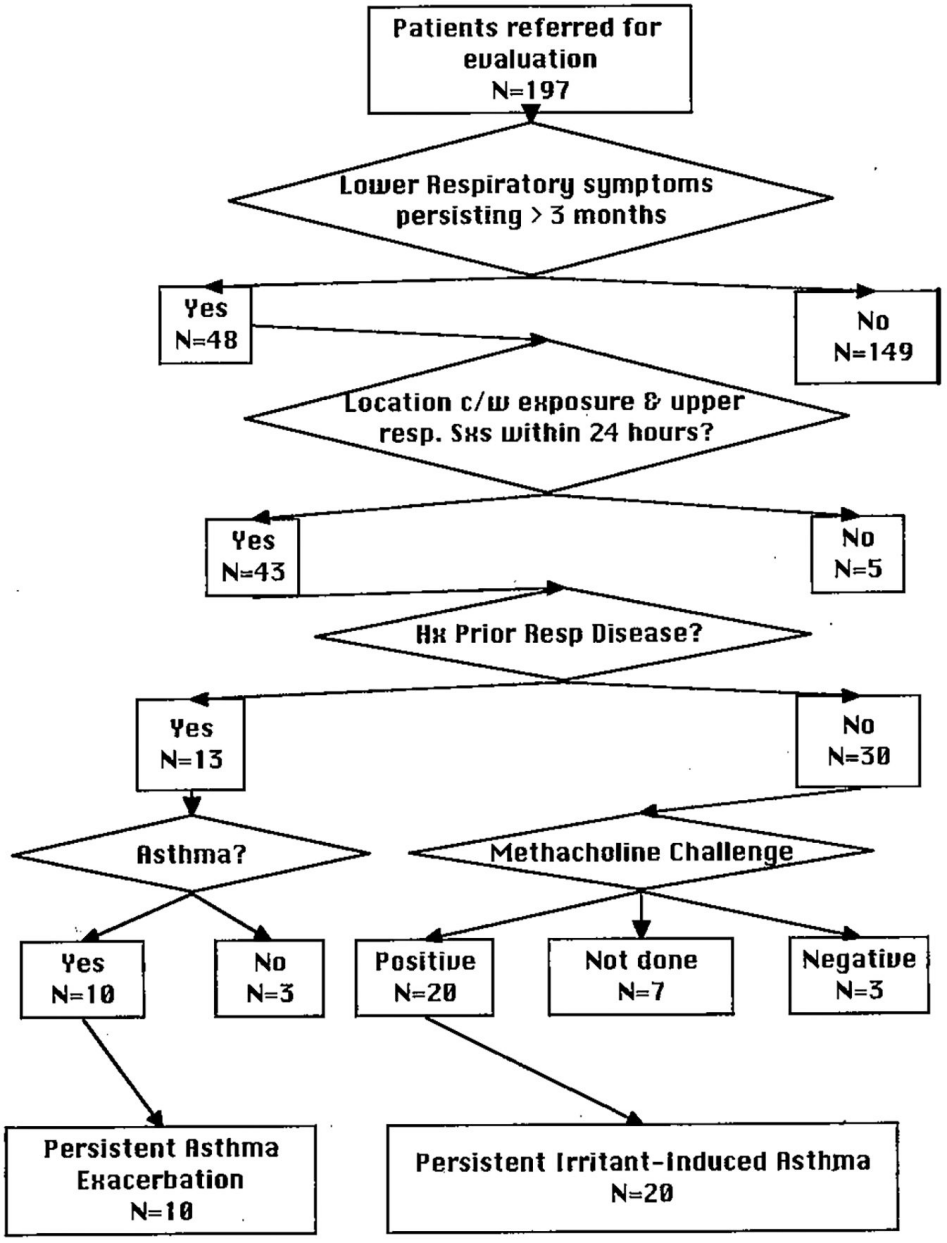
Flow-chart for workup of potential irritant-induced asthma. [Source (54)].

Over a more extended time frame, a total of 197 individuals were referred to medical practitioners in the [San Francisco] Bay Area because of lingering symptoms post-spill and were evaluated by university- affiliated providers. Of those referred, 48 complained of persistent respiratory health complaints for at least three months and consequently underwent a rigorous pulmonary work-up for a possible diagnosis of irritant-induced asthma (53). The latter consisted of a symptom history (and review of medical records), physical examination, and pulmonary function testing including methacholine challenge (a measure of nonspecific bronchial hyperreactivity - Figure 6). In total, 20 of these individuals fulfilled the diagnostic criteria for irritant-induced asthma and another 10 showed persistent exacerbation of pre-existing asthma symptoms (54). The latter publication described itself as “…the first reported series of cases of irritant-induced asthma… affecting community residents as well as workers after a predominantly environmental exposure to a chemical agent.” Subsequent literature review revealed documentation that smaller-scale exposure incidents involving the routine application of Metam sodium to agricultural fields had been linked to respiratory symptoms among downwind residents, consistent with known MITC-mediated effects (55, 56). To our knowledge, there were no reports of long-term ocular complaints related to the MITC gas exposure at Dunsmuir.

Viewed retrospectively, the logistical and technologic resources deployed after the Dunsmuir incident and those at Bhopal a decade earlier differed substantially, making direct comparison of health outcomes difficult between the two events. Although there was considerable overlap in symptom classification, the acute symptoms reported at Bhopal (encompassing individuals interviewed in the first 72 h) cannot directly reflect the fact that an estimated 2,500 individuals did not survive the acute pollution event, nor that for most of these unfortunates their mode of death could – at best – be deduced from autopsy findings. Compounding this deficiency is the fact that in neither situation were direct exposure metrics obtained, and for neither cohort was a demographically matched “control” population identified. Although generalizing our findings to other chemical irritants may prove to be problematic, the juxtaposition of these two incidents provides an opportunity to consider what acute health effects two closely related chemicals (MIC and MITC) may have when a large human population is exposed under uncontrolled conditions (Table 2).

## Comparison of exposure incidents

Symptomatically, acute eye, nose and throat irritation – as well as cough, wheeze and shortness-of-breath – were reported both in Bhopal, India (MIC exposure), and in Dunsmuir, California (MITC exposure). Although hospitalizations occurred in both incidents, the magnitude of the clinical spectrum was dramatically different in Bhopal, extending to severe acute lung damage (progressing in a subset to pulmonary edema and death, with the occurrence of chronic chest symptoms and decrements in lung function in many survivors). By contrast, although most individuals exposed in Dunsmuir reported gradual resolution of symptoms, a subset went on to complain of intermittent respiratory tract symptoms. These included episodic cough, wheeze, chest tightness, and shortness-of breath in response to physical and chemical triggers (consistent with irritant-induced asthma with bronchial hyperreactivity).

One might ask whether this contrast relates to the exposures themselves (i.e., MIC v. MITC), to the state of diagnostic technology at the time of the exposure incidents, or to some combination of these factors. Since the case definition for acute irritant-induced asthma (then referred to as “RADS”) first appeared in the literature in 1985 (*after* the Bhopal spill but *before* the Dunsmuir spill), one can deduce that a technical information gap existed in the published literature, and may have been at least partly to blame for the lack of “clinical suspicion” for RADS in the acute phase of Bhopal.

Epidemiologically, Bhopal also featured more severe eye, nose, throat and chest irritation than was the case in Dunsmuir. The reason for this symptomatic difference quite possibly relates to the properties of the toxicants (MIC having a lower RD_50_ than MITC) as well as differences in exposure dynamics. As noted in Table 1, MIC has a much lower boiling point (and correspondingly higher vapor pressure) than does MITC – and along with its lower RD_50_, – a the more severe irritative symptoms reported in Bhopal were not unexpected.

In terms of exposure dynamics, in the Bhopal incident preformed MIC was released rapidly at ground-level, with still winds and an atmospheric inversion (producing a “choking fog”), whereas in Dunsmuir MITC was generated more gradually in a river valley with relatively steep sidewalls, thereby concentrating the vapors close to the source medium (contaminated river water). Thus high-dose exposures were more common in Bhopal, particularly among those in substandard housing adjacent to the source, or who attempted to escape by running from their homes.

## Consideration of alternative diagnoses

Besides irritant-induced asthma, other potential [irritant-induced] pulmonary diagnoses likely merited consideration, resources allowing. Among these were bronchiolitis obliterans (“BO” – sometimes referred to as “popcorn workers’ lung”) which typically presents with *non-reversible* airway obstruction in small (distal) airways (57, 58). BO can manifest itself after lung or hematopoietic stem cell transplantation, or after exposure to poorly water-soluble gases and vapors (industrial chemicals or commercial flavorants such as diacetyl – (59). Since airway injury in BO occurs in the *distal* portion of the airway, current diagnosis typically pairs pulmonary function results with data from high-resolution computerized tomography (“HRCT”) rather than using transluminal lung biopsy results. Thus, BO diagnosis came to depend upon a technology which – although initially referenced for pulmonary use as early as 1982 – was not widely available during the immediate post-Bhopal incident response. Similarly evasive diagnoses may have included subacute (or chronic) chemical bronchitis and various forms of interstitial fibrosis.

## TRPA1 – structure, function and pathophysiologic relevance

Early in our discussion, we emphasized the importance of TRPA1 as a *shared link* in the biological response to MIC and MITC (two irritants which can only be described as “*first cousins*”). We also mentioned that this ion channel responds to a *wide range* of chemical ligands. In the following paragraphs we will expand on this theme and hopefully reinforce the emphasis we have placed on this ion channel.

In general terms, when studying chemically induced mucous membrane/respiratory tract symptoms, attention naturally focuses on the receptors involved in sensing the initial chemical contact. The term “nociceptor” (“noci…” from noxious and “…ceptor” from receptor) was coined early in the 20th century and has persisted in contemporary usage to describe receptors transducing the sensations of pain, burning, itching, noxious temperature, and pH extremes (60). Secondarily, nociceptor stimulation can trigger a variety of reflex responses, ranging from obvious motor/behavioral (e.g., escape behavior), to biochemical (e.g., autonomic responses such as rhinorrhea and airway congestion), and finally molecular biologic responses. Historically, characterization of nociceptors has most often considered to have reached a landmark with the explicit biochemical characterization (or “cloning”) of receptor protein sequences.

**Figure 7:**
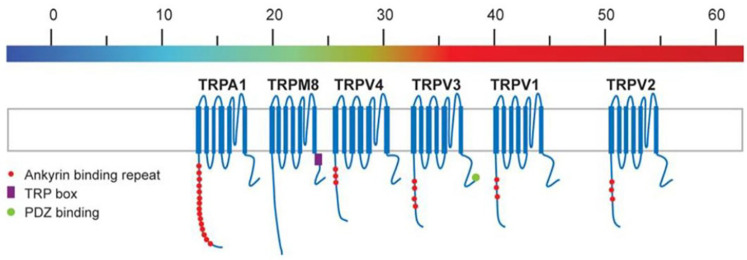
Thermosensitive TRP ion channels arranged in an array are thought to serve as a “molecular thermometer” *in vivo*. [REF (61)].

Human nociceptor cloning first generated attention with the sequencing of nicotinic and purinergic receptors (in the early 1980's), followed by acid-sensitive ion channels (early 1990's) and more recently the tetrameric ion channels combining chemical- and temperature-sensing (late 1990's onward). Selectively referencing these latter receptors by their “prototype” ligands, cloning of TRP ion channels began with TRPV1 (the capsaicin receptor - 1997), followed by TRPM8 (menthol - 2002), and finally TRPA1 (allyl isothiocyanate – also referred to as the “mustard oil” or “wasabi” receptor - 2004). During this time, it was further realized that most TRP channels respond to *multiple* physical and chemical stimuli, with TRPV1 responding to capsaicin, acid (H^+^) and heat (> 42 ^o^ C); TRPM8 to menthol + cold (<25°C); and TRPA1 to allyl isothiocyanate + noxious cold (< 17^o^ C). Conveniently, the above spread of TRP receptor temperature sensitivities lends them the ability to act collectively as an *in vivo* “molecular thermometer” (Figure 7).

**Figure 8:**
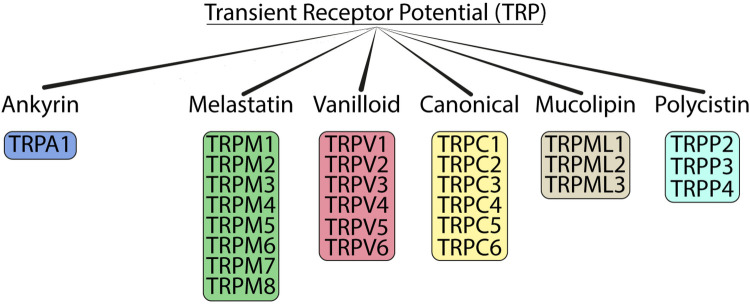
Organization of human TRP ion channel families. TRPA1 is the sole member displaying only a single isoform. Source (62): – Creative Commons 4.0.

**Figure 9:**
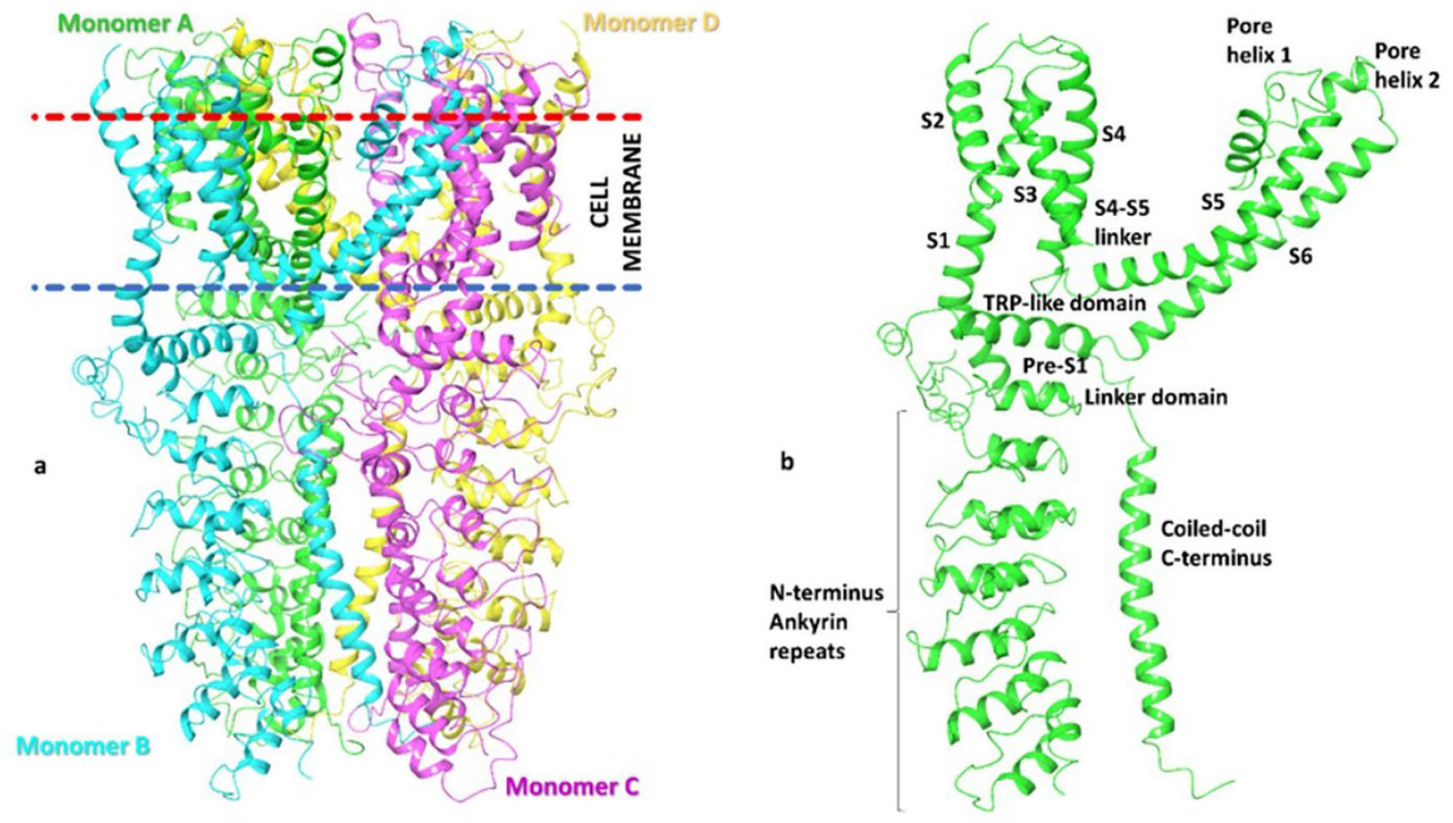
Structure of aggregate TRPA1 receptor. **(a)** Arrangement of homotetramer array; **(b)** Substructures involved in each monomer [Source (63); CC BY 4.0].

Given this background, we can ask: *What – if anything – is unique about TRPA1, and how does it relate to TRPV1, with which it is frequently co-located in C- and Aδ- fibers?* Aside from its shared role in sensing irritants at Bhopal (MIC) and Dunsmuir (MITC), the TRPA1 receptor displays several unique features, including:

1. It is the sole human TRP channel for which only a single isoform exists (Figure 8).
2. The receptor itself consists of a trans-membrane homotetramer variably permeable to calcium and sodium ions, with an aggregate molecular weight of 468 kDa (Figure 9). The receptor can activate at very low (peri-threshold) concentrations if TRPV1 receptors are co-present and also being stimulated (64).
3. The N-terminal region of each monomer contains a large number of repeating ankryn (polypeptide) residues (*n* = 16), several times the number in its nearest competitor among TRP ion channels. It has been suggested that this configuration may possibly hold the key to the wide range of ligands to which TRPA1 responds (65, 66).
4. Although TRPA1 is frequently co-localized with TRPV1 on unmyelinated c-fibers, it can respond to selected electrophiles (e.g., acrolein; hypochlorite) and solvents (e.g., styrene) *without* the functional participation of TRPV1 [(6), page 1903].
5. Although virtually all chemosensitive TRP channels respond to chemical stimuli beyond their prototype ligand, TRPA1 is notable for its sheer number of chemical ligands (Table 3).

**Table 3:** Representative chemical ligands known to activate TRPA1.

Chemical class	Example	Chemical class	Example
Aldehydes (*α*, *β* unsaturated)	Acrolein Crotonaldehyde Cinnamaldehyde Super cinnamaldehyde	Aldehydes (saturated)	Formaldehyde Acetaldehyde
Sulfur compounds	Allicin Sodium bisulfite Diallyl disulfide Sulfur mustard	Alcohols	Menthol Thymol Carvacol
Reactive oxygen species	Ozone Hydroxyl radical Hydrogen peroxide Hypochlorite	Reactive nitrogen species	Chloropicrin Chlorobenzyl malonitrile
Cannabinoids	*Δ*^9^ Tetrahydrocannabinol Cannabigerol Cannabidiol Cannabigeroquinone Cannabidiolic acid Cannabichromene Phytocannabinol	Tear gas agents	Benzyl bromide Bromoacetone Phosgene Chloropicrin 2-chloroacetophenone 2-chlorobenzalmalononitrile Dibenz [bf] [1,4] oxazepine
Isocyanates & isothiocyanates	Methyl isocyanate Methyl isothiocyanate Allyl isothiocyanate Toluene diisocyanate	Prostaglandins	Prostaglandin A2 15-deoxy-Delta(12,14)- prostaglandin J (2)

Compounds in red represent toxicants released (or formed) at Bhopal or Dunsmuir.

## TRPA1 toxicology – agonists and antagonists

Stimulation of TRPA1 receptors (by a variety of ligands) can trigger such sensations as pain, itch, lacrimation, vasodilation, hypersecretion, sneeze, cough, and bronchospasm (67, 68). Since TRPA1 is – in general – expressed on *sensory* afferents, the mechanism(s) whereby these symptoms occur likely include some combination of the following:

• Direct sensory stimulation (localized pain)

• Local neuropeptide release (“antidromic” reflex)

• Activation of inflammatory pathways via local release of cytokines,

• Modulation of immune cell function (including mast cells)

• Oxidative stress

• Participation in sensory-motor reflexes (including coughing, sneezing, and bronchospasm).

In terms of TRPA1 agonists, it is important to distinguish between plant-derived “mustard oil” (allyl isothiocyanate, the prototype TRPA1 ligand) and “sulfur mustard” (2-chloroethyl ethyl sulfide, both a TRPA1 ligand *and* an alkalating agent utilized for chemical warfare in World War I.) Other studies highlight distinct aspects of the receptor's response. For example, both diesel exhaust particles (“DEPs”) and sidestream tobacco smoke (“STS”) have TRPA1-stimulating activity. Both substances contain a significant component of polycyclic aromatic hydrocarbons or “PAHs,” which can act as agonistic electrophiles (86). PAHs are also present in woodsmoke, exposure to which can increase the expression of MUC5AC in both the upper airways (as in rhinitis) and lower airways (as in asthma) (69, 70). Persuant to this diversity, Table 3 hints at the multitude of TRPA1 ligands that have been identified.

Reciprocally, we could also ask whether TRPA1 receptor-specific *antagonists* offer *protection* from adverse effects if given prior to (or promptly after) exposure? In a study of “knockout” mice (genetically deficient in TRPA1), the anticipated noxious effects of three different tear gas compounds were averted post stimulus-challenge. In that same study, adverse effects were also averted (both *in vitro* and *in vivo*) by pre-administration of either of two proprietary TRPA1 antagonists (HC-030031 or AP-18). Mice pre-treated with either of these two agents prior to intraconjunctival or hindpaw injections showed no response to the application of TRPA1-specific irritants (71). In another study published that same year, Caceres and colleagues (72) examined the respiratory tract effects of exposing TRPA1 knock out mice (as well as mice pre-treated with HC-030031) using an inhaled allergen to which the animals had previously been sensitized. Both genetic ablation and pharmacologic inhibition of TRPA1 inhibited allergen-induced leukocyte airway infiltration, cytokine and mucus production, and airway hyperreactivity. TRPA1 -/- mice also showed deficiencies in neuropeptide release, potentially explaining TRPA1's permissive role in asthmatic inflammation. A similar respiratory response inhibition was observed when ovalbumin-sensitized guinea pigs were pretreated with BIO 1305834 [yet another TRPA1 antagonist; (73)]. Other apparent TRPA1 antagonists include A-967079, GRC 17536, and GDC-0334, as well as cannabidiol, capsazepine, and FAAH/COX-2 inhibitors, each of which can block selected TRPA-1 mediated adverse symptoms.

Table 3 (see above) not only highlights the fact that both MIC and MITC are TRPA1 ligands, but also that a host of other commonly encountered chemicals share that property. For example, listing of “hypochlorite” under reactive oxygen species highlights the active portion of the chemical we often refer to simply as “bleach” (which is one of the most commonly used surface disinfectants in homes and commercial settings). Hypochlorite appears to be able to induce airway hyperreactivity by interacting with TRPA1 alone, absent TRPV1 [(74), p. 1903]. Occupations at-risk of respiratory symptoms from bleach include house and commercial cleaners, health care workers, and others (75, 76). Chlorine-based disinfectants are also used in water purification (including in swimming pools), which is linked, in turn, to the fact that swimming pool workers (e.g., lifeguards and attendants) are likewise at risk of respiratory effects, independent of known allergies (77, 78). If, indeed, TRPA1 alone can mediate the development of substance-specific sensory hyperreactivity (e.g., to hypochlorite), then a case could be made that the ion channel itself is *mimicking a specific allergic response* (79).

## Epidemiologic “take-home” messages from Bhopal and Dunsmuir

Retrospective examination of the Bhopal incident suggests that a model of acute + persistent damage to small conducting airways is in order. Consistent with this, several studies alluded to in the Bhopal section referenced intraluminal desquamation of cellular debris in the lungs of experimental animals (58). An epidemiological study done by Beckett (80) references “persistent small airways obstruction” as being attributable to [MIC] gas exposures. In general, then, both animal and human data supported the potential for MIC to cause small airway disease (including possible bronchiolitis obliterans) and may justify the use of HRCT to screen exposed individuals with persistent symptoms.

Regarding potential RADS induction (i.e., in Dunsmuir), Brooks' clinical algorithm was clearly of great utility, and by contrast – as Nemery (20) pointed out – the fact that measures of nonspecific bronchial reactivity were never systematically obtained on members of the Bhopal cohort renders their longitudinal studies the status of a “lost experiment.”

## Overall discussion and conclusions

This review set out to examine two mass-casualty incidents and to retroactively explore the potential role of the TRPA1 receptor therein. In doing so, we learned that TRPA1 has a very wide range of ligands, some of which can potentially stimulate nerve fibers (and trigger reflexes) without the involvement of other (non-TRPA1) nociceptive ion channels. Our two mass exposure incidents (Bhopal and Dunsmuir) had in common – not only near-identical toxicants (MIC and MITC) – a but also the induction of mucous membrane (eye, nose and throat) as well as chest irritation (albeit with differing sequelae). At Bhopal, there was a significant incidence of chemical pneumonitis/non-cardiogenic pulmonary edema, with many early fatalities in heavily exposed individuals, plus a considerable prevalence of chronic small airway obstruction lasting years thereafter. In addition, significant acute (and largely reversible) ocular pathology was evident post-exposure.

At Dunsmuir, on the other hand, acute irritative symptoms were – for the most part – less severe (although some individuals with pre-existing obstructive respiratory tract conditions were briefly admitted as inpatients to local hospitals). Among roughly 200 exposed individuals with persistent symptoms who eventually found their way to occupational medicine specialists in the San Francisco Bay area, nearly 50 reported the new onset of intermittent chest tightness, wheeze, and shortness of breath, the then-newly developed criteria for “RADS” (acute-onset irritant-induced asthma) were applied. Ultimately twenty of these individuals satisfied the diagnostic criteria for RADS, and another ten manifested objective exacerbations of pre-existing asthma (54).

Reflecting the evolving state of medical knowledge and expertise, different approaches ended up being applied at different times between these two mass casualty investigations. The diagnostic criteria for irritant-induced asthma were not published until the year after the Bhopal incident, rendering it useful for analyzing the Dunsmuir – but not necessarily the Bhopal – incident. Similarly, the diagnosis of “bronchiolitis obliterans” (non-reversible small airway obstruction), while a theoretical possibility when Bhopal occurred, was predicated on the use of a technology (“high-resolution computerized tomography”) which by 1984 was only highlighted in a single published respiratory study (58). Other potential lung responses to oxidative stress – including subacute/chronic chemical pneumonitis and interstitial fibrosis (more prevalent with autoimmune conditions or dust inhalation) would all figure into a complete differential diagnosis of persistently symptomatic individuals exposed at Bhopal, given appropriate knowledge, tools and resources.

Looking forward, pretreatment (or prompt post-exposure treatment) with emerging TRPA1 antagonists has been shown to blunt the response to irritants in experimental animals and may become candidates for future human trials. Although we have largely confined our discussion to exogenous ligands for TRPA1, researchers also continue to identify novel *endogenous* ligands that stimulate this ion channel. Thus, TRPA1 function my turn out to be of relevance – not only to physiology and toxicology– but also to the practice of allergy, rheumatology, gastroenterology, and other branches of medicine (81). Hopefully, this (and subsequent) discussions can help foster a deeper appreciation of the TRPA1 receptor (with its many exogenous and endogenous ligands) along with its potential contributions to the development of chronic airway conditions such as rhinitis, asthma, bronchitis, bronchiolitis obliterans, and interstitial fibrosis.

## Appendix A

**Table A1:** Characteristics of exposure incidents.

Exposures	Bhopal	Dunsmuir
Predominant exposure	Methyl isocyanate (MIC)	Methyl isothiocyanate (MITC)
Source of toxicant	Pre-formed (in tank)	In Situ (in river)
Exposure route	Inhalation/eye	Inhalation/eye/ ± dermal
Exposure duration	<24 h	≤ 4 days
Peak air exposure levels	≤25,000 ppb (estimated)	≤1,600 ppb (estimated)
# Exposed	∼ 200,000	>700
Acute hospitalizations	>>1,000 (crude estimate)	7
Acute mortality	∼ 2,500	0

Singh and Ghosh (12).

Alexeeff et al. (45).

## Appendix B

**Table B1:** Long-term studies of Bhopal-related respiratory symptoms and signs.

Author	Design	#	Time*	Findings & limitations
Kamat et al. (82)	Case-series	113	2 yrs	Minimal symptom improvement over 24 months. 58% improved CXR over 24 months. Decreasing FEV_1_/FVC (but increasing peak flow) w/time. Inflammatory exudates in small airways.
Vijayan and Sankaran (22)	Case-series	60	1–7 yr	Decreased FEV1/FVC with higher exposures ^ BAL macro ϕ & lymphocytes with higher expos. 11/41 Obstructive PFTs in high-exposure group 8/41 Restrictive PFTs in high-exposure group
Cullinan et al. (83)	Cross-sectional	474 Sxs	10 yr	^ Respiratory symptoms (81% v 38%) in high- exposure group
Cullinan et al. (23)	Cross-sectional	454 Sxs 74 PFTs	10 yr	Symptom prevalence decreased with increasing distance from plant. PFTs (particularly FEF_25−75_) decreased among those most closely exposed to the plant.
Dhara and Dhara (84)	Cross-sectional	312 Sxs 74 PFTs	10 yr	Cough, asthma, shortness-of-breath & decreased FEF_25−75_ most closely related to “total exposure weighted for distance.” Suggestion that current dyspnea might reflect irritant-induced asthma but *does not suggest or utilize methacholine challenge to test this hypothesis*.
De et al. (85)	Cross-sectional - sequential	4,304	32 yr	Cumulative analysis of cohort data (initial cohort, ∼78,000 in 1986, decreasing to ∼17,000 in 2007, & rebounding with recruitment to ∼31,000 in 2016). Exposure zone-related symptoms (dyspnea, wheeze, cough, sputum production) evident in all exposed age groups, but normalizing with time. Acute impairment resolving over ∼15 years was most evident among those exposed as children (<10 years) and teenagers (>10 and <20 years).

“Time” = duration of follow-up.
